# Thermally Assisted Optically Stimulated Luminescence (TA-OSL) from Commercial BeO Dosimeters

**DOI:** 10.3390/ma16041494

**Published:** 2023-02-10

**Authors:** Georgios S. Polymeris

**Affiliations:** Laboratory of Archaeometry, Institute of Nanoscience and Nanotechnology, National Centre for Scientific Research “Demokritos”, 15310 Agia Paraskevi, Greece; g.polymeris@inn.demokritos.gr

**Keywords:** BeO, TL, TA-OSL, supralinearity, thermally assisted processes

## Abstract

BeO is another luminescent phosphor with very deep traps (VDTs) in its matrix that could not be stimulated using either thermal or conventional optical stimulations. The present study attempts to stimulate these traps using thermally assisted optically stimulated luminescence (TA-OSL), a combination of simultaneous thermal and optical stimulation that is applied to the material following a thermoluminescence measurement up to 500 °C. An intense, peak-shaped TA-OSL signal is measured throughout the entire temperature range between room temperature and 270 °C. This signal can be explained as the transfer of charges from VDTs to both dosimetric TL traps. Experimental features such as the peaked shape of the signal along with the presence of residual TL after the TA-OSL suggest that recombination of TA-OSL takes place via the conduction band. Isothermal TA-OSL is not effective for extending the maximum detection dose thresholds of BeO, unlike minerals such as quartz and aluminum oxide. Nevertheless, TA-OSL could be effectively used in order to either (a) control the occupancy of VDTs, circumventing the intense sensitivity changes induced by long-term uses and high accumulated dose to the VDTs, or (b) measure the total dose accumulated over a series of repetitive dose calculations.

## 1. Introduction

Beryllium oxide (BeO) could be considered as the luminescence dosimeter of the current decade. This material was very early suggested, during the 1960s, as a thermoluminescence (TL) dosimeter, due to a plethora of advanced properties. The most important include an easy TL glow curve with well-isolated, non-overlapping TL peaks, increased sensitivity to ionizing radiation, linearity of dose–response, and a near-tissue equivalent value of Z_eff_ = 7.14, i.e., very close to the value of Z_eff_ = 7.35–7.65 for biological tissues and the value of Z_eff_ = 7.51 for water [1]. This means that this material has a minimal (<20%) over- and under-response to low-energy photons, which is optimal for a wide range of applications in different fields of medicine, such as radiotherapy or radiation diagnostics [2,3,4]. A typical TL glow curve of a BeO dosimeter yields three TL glow peaks, with temperatures *T_m_* corresponding to maximum intensities *I_m_* at ~75, 190, and 317 °C for heating rate 1 °C/s [5]. 

Based on the number of available TL peaks within a TL glow curve and their corresponding peak positions, an experimental TL glow curve is usually divided into three main temperature regions [6]. Starting from room temperature (RT) up to around 150 °C, this region is considered the low-temperature region, including shallow traps. These traps are thermally unstable at ambient temperatures and thus inappropriate for dosimetric applications. The TL peak at 75 °C stands as a typical example of a TL peak at this specific region. Ranging between 150 and 300 °C, the second temperature region is considered the main dosimetric region. These traps are thermally stable at ambient temperatures and thus appropriate for dosimetry applications. All commercially available TL dosimeters, such as beryllium oxide, yield TL peaks at this specific temperature region [7]. The TL peak at 190 °C is the main dosimetric TL peak of BeO, as it is the TL peak that is widely used for dosimetric applications. Finally, the temperature region ranging between 300 and 500 °C is called the high-temperature region, corresponding to relatively deeper traps. These traps yield very prolonged lifetimes and are consequently more appropriate for dosimetry using natural phosphors, especially for dating applications; the TL peak with delocalization temperature *T_m_* at 317 °C is a typical example. 

However, the increased sensitivity of the material to light was firstly recognized as a major drawback that recently has resulted in a new advantage; BeO was suggested as a sensitive dosimeter for Optically Stimulated Luminescence (OSL). Bulur and Göksu [8] were the first who reported new (OSL) properties from an old dosimetric (TL) material before it was finally adopted in a commercial OSL dosimetry system [2,9,10,11,12]. The majority of the aforementioned TL advantages are relevant also for the OSL signal, in conjunction to a convenient OSL shape that yields a fast-decaying OSL component [13,14,15]. Nevertheless, the mechanism responsible for the OSL signal is not completely understood. Thus, conflicting results exist regarding the origin of the trapped charges responsible for the OSL signal. Recently, the low (5%), albeit substantial, fading in the OSL signal in the first 24 h of storage in the dark at room temperature, followed by the stability of the signal over many months, was recognized as another disadvantage. Among several other TL and OSL features, this latter quality was thoroughly discussed in the framework of the latest Solid-State Dosimetry conference that took place in Hiroshima, Japan in 2019. For an overview on both favorable and limiting properties of BeO, readers are referred to recent articles on the topic by Aşlar et al. [14], Polymeris et al. [15], and Yukihara [3,16,17].

Among these properties, intense transfer effects were often reported in the luminescence signal from BeO. Bulur and Göksu [8] were the first authors to report intense the OSL signal following TL without any intermediate irradiation. According to their terminology, they have measured “OSL after TL” at room temperature. This is exactly the thermally stimulated recuperation of the OSL signal that Yukihara [3,16] later termed thermally transferred OSL (TT-OSL hereafter). This signal was detected following previous TL within a narrow temperature range (225 to 310 °C), with a maximum intensity of barely 50% of the corresponding OSL intensity at 275 °C. Moreover, the TT-OSL curve indicates the typical decaying shape to that of a trivial OSL signal, which suggests that the same trapping centers are responsible for the OSL and the TT-OSL signal [3,16]. In an effort to exploit this specific signal for dosimetric applications, Yukihara has reported dose–response features for both low- [3] and high-dose [16] regions. Phototransfer is another proof of transfer effect that is being observed from stable trapping centers in BeO; it is stable enough to be occupied after heating to temperatures higher than 400 °C, leading to either TL (PTTL [18]) or OSL signals (PTOSL [19]).

Intense transfer effects were also reported by Aşlar et al. [14] in their effort to study possible correlations among the TL, OSL, and Electron Paramagnetic Resonance (EPR) signals in commercially available BeO dosimeters. Transfer was monitored either (a) between the two dosimetric traps or (b) from deeper than both traps responsible for the dosimetric TL peaks. Due to intense phototransfer effects from deeper to shallower traps, quantitative correlation using the signal intensities as a unique probe was not recommended. Moreover, following deconvolution of OSL decay curves and reconstruction, namely correction for thermal quenching, these authors have reported OSL components indicating an integrated signal that increases with increasing measurement temperature. This is a characteristic property of a thermally assisted process. Thus, Aşlar et al. [14] have suggested that indirect thermally assisted optically stimulated luminescence (TA-OSL) signals were monitored, indicating that transfer is more intense towards the trap corresponding to the TL peak at 317 °C. For this reason, even after depleting both TL peaks, intense OSL curves were still measured.

TA-OSL comprises simultaneous thermal and optical stimulation of traps that indicate delocalization temperatures *T_m_* higher than 500 °C; these were termed very deep traps (VDTs hereafter). According to the early publication by Polymeris et al. [20], initially all conventional traps are emptied by a TL measurement up to 500 °C. Then, TA-OSL is measured in a continuous wave mode at a high temperature, promptly after the TL measurement and without any additional dose; it is the isothermal TA-OSL mode [6]. The signal registers the charge that was captured at traps with activation energies beyond 2 eV [6] that is transferred to shallower, albeit dosimetric, traps due to being empty because of the previous TL measurement. In other words, it is the most appropriate technique for stimulating VDTs, without heating the dosimeter to temperatures higher than those imposed by luminescence instrumental limitations [21,22]. The presence of such very deep traps is almost a ubiquitous feature of all wide-band semiconductors, either naturally occurring such as quartz, feldspars, and NaCl, or artificial such as Al_2_O_3_ with various dopants, BaSO_4_:Eu, etc.; for an extended list of these materials, readers are referred to [22]. In this latter publication, just a selection of TA-OSL curves is presented for the case of BeO; nevertheless, a detailed study on the dosimetric properties of this signal is still missing from the literature. 

TA-OSL was initially suggested as an alternative experimental method in order to not only measure the signal of the much deeper traps in Al_2_O_3_:C without heating the sample to temperatures greater than 500 °C, but also use this signal for high-dose-level dosimetry purposes as well [20]. Later on, TA-OSL was identified as a promising technique for extending both luminescence dosimetric [6] and dating limits [23], due to the following assets: (a) the lifetimes of such traps, of the order of 10^9^ years or even more [6], combined with (b) insignificant athermal (anomalous) fading losses, even for the cases of reference materials such as feldspars or apatites [24,25], and (c) the higher charge capacity of these traps, resulting in dose–response curves of favorable features and larger dose saturation levels. Minerals such as quartz [26,27] and Al_2_O_3_:C [20,21,22] are being extensively studied, providing promising TA-OSL features and properties. Nevertheless, the favorable properties of TA-OSL for either dating or dosimetric applications are not reported for all materials. Typical examples include NaCl [28] and MgO (under preparation). 

The present work attempts to fill in the hiatus of the scientific literature regarding the lack of an extended TA-OSL study in commercially available BeO samples. The aim of the present work is multifold, including (a) a detailed study on the TA-OSL features and properties of commercial BeO, (b) optimization of the measuring parameters, (c) study on the dose–response properties of the corresponding signal, and (d) comparison of the dose–response features of both conventional photo-transferred TL.

## 2. Materials and Methods

### 2.1. Materials and Apparatus

Commercially available BeO dosimeters in the form of pellets were used in the framework of the present study. The pellets were in square disc form with dimensions of 4 mm and thickness of 1 mm and were purchased from Thermalox 995, Brush Wellman Inc., USA. These detectors were annealed in an oven, inside an alumina crucible, at 900 °C for 60 min before the experiments, cooled immediately by putting the crucible in contact with a heat sink. The temperature was selected in order to empty all traps. 

All stimulated luminescence measurements were performed using a Risø TL/OSL reader (model TL-OSL-DA-20, Reader ID: 267) equipped with a (0.12 ± 0.03) Gy/s ^90^Sr/^90^Y β-ray source. Unless otherwise stated, all TL and RTL measurements were performed up to a temperature of 500 °C. In all cases, a low heating rate of 2 °C/s was applied to avoid significant temperature lag [29]. The OSL measurements were performed in the continuous wave (CW-OSL) mode with blue LEDs (~470 nm) at constant stimulation intensity (90% of the maximum 36 mW/cm^2^). All stimulated luminescence signals were recorded using a 7.5 mm thick Hoya U-340 filter (270–380 nm, FWHM 80 nm) in front of a bi-alkali EMI 9235QB photo multiplier tube (PMT). The test dose for each protocol is different.

### 2.2. Experimental Protocols

The experimental protocols that were applied constitute modified versions of the protocols that have been previously used for the study of TA-OSL in the cases of Al_2_O_3_:C [21] as well as of NaCl [28]. Two major protocols were applied, one for the selection of the appropriate stimulation temperature of the TA-OSL measurement, along with one that studies the dosimetric properties of the corresponding signal. In the former case, TA-OSL is measured for various stimulation temperatures according to protocol A (Table 1). In the latter case, the TA-OSL signal is measured for various doses to check the dose–response behavior of the signal. The two different protocols include steps for checking the sensitivity of the dosimetric traps of the material, following emptying the VDTs. Sensitivity is measured as the integrated TL peak signal following a steady test dose; a different test dose was used for each protocol. In addition to the action involved for each step, comments on the corresponding necessity of each step within the protocols are detailed in Table 1 and Table 2. Each cycle of measurements was executed for both protocols using a different, freshly annealed BeO disk.

### 2.3. RTL Deconvolution and PSM

All RTL glow curves were analyzed into individual TL peaks using the analytical expressions in the framework of the one trap one recombination center (OTOR) model [30]. The analytical expression given by Kitis and Vlachos [31] takes advantage of the Lambert W function *W*(*z*) [32]:

(1) $$I\left(T\right)=I_{m}\exp\left(-\frac{E}{kT}\cdot \frac{T_{m}-T}{T_{m}}\right)\cdot \frac{W\left(z_{m}\right)+W{\left(z_{m}\right)}^{2}}{W\left(z\right)+W{\left(z\right)}^{2}}\;$$

where *z* is now approximated by the expression:

(2) $$z=\exp\left(\frac{R}{1-R}-\ln\left(\frac{1-R}{R}\right)+\frac{E\cdot \exp\left(\frac{E}{kT_{m}}\right)}{kT_{m}^{2}\left(1-1.05\cdot R^{1.26}\right)}\cdot F\left(T,E\right)\right)$$

and *z_m_* is the value of *z* from Equation (2) for *T = T_m_*.

Here *R* denotes the ratio *A_n_/A_m_*, with *A_n_* and *A_m_* being the re-trapping and recombination coefficients, respectively (in cm^3^ s^−1^). The *p*(*t*,*T*) corresponds to the stimulation probability which is described by Equation (3):

(3) $$p=\tau^{-1}=s\cdot \exp\left(-\frac{E}{kT}\right)$$

Equations (1)–(3) are used to fit the experimental TL glow curves, with the activation energy *E*, the temperature of maximum intensity *Tm*, and the ratio *R* (with R < 1) being the fitting parameters. The function *F*(*T*,*E*) in this expression is the exponential integral appearing in TL models and is expressed in terms of the exponential integral function *E_i_*[*−E/kT*] as [30,32,33]:

(4) $$F\left(T,E\right)=\int_{T_{0}}^{T}e^{-\frac{E}{kT}}dT=T\cdot \exp\left(-\frac{E}{kT}\right)+\frac{E}{k}\cdot E_{i}\left[-\frac{E}{kT}\right]$$

There are two numerical methods to evaluate the exponential integral function *E_i_*[z]: (a) as an elementary function in commercially available software packages and (b) through approximate analytical expressions. For the majority of the cases, it has been implemented as an elementary built-in function in various software packages [30]. 

All curve fittings were performed using the software package Microsoft Excel, with the Solver utility [34], while the goodness of fit was checked using the Figure Of Merit (F.O.M.) [35]:

(5) $$F.O.M.=\sum_{i}\frac{\left|Y_{\exp}-Y_{\mathrm{Fit}}\right|}{A}$$

where $Y_{\exp}$ is the data point on the experimental curve, $Y_{\mathrm{Fit}}$ is the data point on the fitted curve, and *A* is the area of the fitted curve. A successful deconvolution process is obtained by optimizing fitting parameters so that *F*.*O*.*M*. values are as low as possible. For the present study, *F.O.M.* values were lower than 3.1%. 

The peak shape methods are used in order to calculate the activation energy of the two RTL peaks, as these are not overlapping but isolated. The peak shape methods take advantage of the geometrical characteristics of the RTL glow curve, mainly the delocalization peak temperature *T_m_* and the high- and low-temperature (T_1_ and T_2_, respectively) sides of the glow curve in the half maximum intensity. The approach of Kitis and Pagonis [36] was adopted, based on the general order kinetics model. Thus, the presented calculations in this work are based on the following equations:

(6) $$C_{\omega }=\frac{\omega \cdot {Ι}_{m}}{\beta \cdot n_{0}}$$

(7) $$C_{\delta }=\frac{\delta \cdot {Ι}_{m}}{\beta \cdot n_{m}}$$

(8) $$C_{\tau }=\frac{\tau \cdot {Ι}_{m}}{\beta \cdot (n_{0-n_{m}})}$$

In these equations $I_{m}$ expresses the maximum TL intensity, β is the heating rate, $n_{m}=\int_{T_{m}}^{\infty }I\;dt$ is the concentration of trapped electrons at maximum (half integral at high-temperature maximum, in units of ${\mathrm{cm}}^{-3})$, $n_{0}=\int_{0}^{T_{max}}I\;dt$ is the initial concentration of trapped electrons (total integral, also in units of ${\mathrm{cm}}^{-3})$, and $C_{\omega },\;C_{\delta },\;C_{\tau }$ are the quantities that characterize the degree by which the area of a single glow curve approaches the area of a triangle. The following geometrical quantities can be defined:

(9) $$\delta ={Τ}_{2}-{Τ}_{m},\;\tau ={Τ}_{m}-{Τ}_{1},\;\omega ={Τ}_{2}-{Τ}_{1}\;\mu_{g}=\delta /\omega$$

Here $\;\mu_{g}$ defines the symmetry (or geometrical) factor of the TL glow peak. The estimation of the activation energy is based on the ω and the integral symmetry factor, which can be defined as the ratio of the concentration of trapped electrons at maximum ($n_{m}$) over the initial concentration of trapped electrons ($n_{0}$), thus $\mu_{g}^{\prime }=\frac{n_{m;}}{n_{0}}$; therefore, according to [31,36,37], Equations (6)–(8) could be expressed as:

(10) $$E_{a}=C_{a}\cdot \left(b^{\frac{b}{b-1}}\right)\cdot \frac{k\cdot T_{m}^{2}}{a}-2kT_{m}$$

where α can be ω, δ, or τ.

In Equation (2) of the OTOR model, the value of the fitting parameter *R* denotes whether the re-trapping probability is significant; for values close to zero, the re-trapping probability is insignificant, while for values close to one, recombination and re-trapping probabilities are comparable. Similarly, in Equation (10), parameter *b* signifies the order of kinetics in the corresponding GOK models; for *b* values close to unity, the first order of kinetics implies negligible re-trapping, while for values close to two, the second order of kinetics suggests significant re-trapping probability. 

### 2.4. Thermal Quenching and Reconstruction

As TA-OSL measurements include the combined action of thermal and optical stimulation, the presence of the thermal quenching effect is expected to substantially suppress the TA-OSL intensity. Thermal quenching describes the decrease in luminescence efficiency with increasing temperature [38] due to the increased probability of non-radiative transitions. The dependence of the luminescence efficiency, η(T), on the temperature is given according to the following formula [38]:

(11) $$\eta \;\left(T\right)=\frac{1}{1+Ce^{-\frac{W}{k\;T}}\;}$$

where the parameters *C* and *W* are the so-called “quenching parameters”, *T* is the temperature in units of K, and k is the Boltzmann constant. For more details regarding the physical meaning of the *W* parameter (in units of eV) as well as the dimensionless *C* parameter, readers are referred to [39]. For the case of isothermal TA-OSL, as the entire measurement takes place at a steady temperature ***T_st_***, the corrected unquenched integrated TA-OSL intensity *I_uq_*(***T_st_***) can be calculated using the following formula:

(12) $$I_{uq}\left(Tst\right)=\frac{I_{q}\left(Tst\right)}{n\left(Tst\right)}$$

where *I_q_(****T_st_****)* and *I_uq_(****T_st_****)* are the integrated TA-OSL intensities which are quenched and unquenched, respectively, while *η*(***T_st_***) corresponds to a single value of the thermal quenching efficiency at the specific measurement temperature. In this study, reconstruction was applied according to Equation (11), using the values of thermal quenching parameters *W* and *C* obtained by Aşlar et al. [40] for the Hoya U340 filter; the specific values used were W = 0.62 eV and C = 1.3 × 10^6^, namely, the values that were calculated for TL peak 2. These values were used because the Hoya U-340 filter was used for all measurements.

## 3. Results

### 3.1. Shapes of TL, TA-OSL, and RTL Glow Curves 

**Figure 1:**
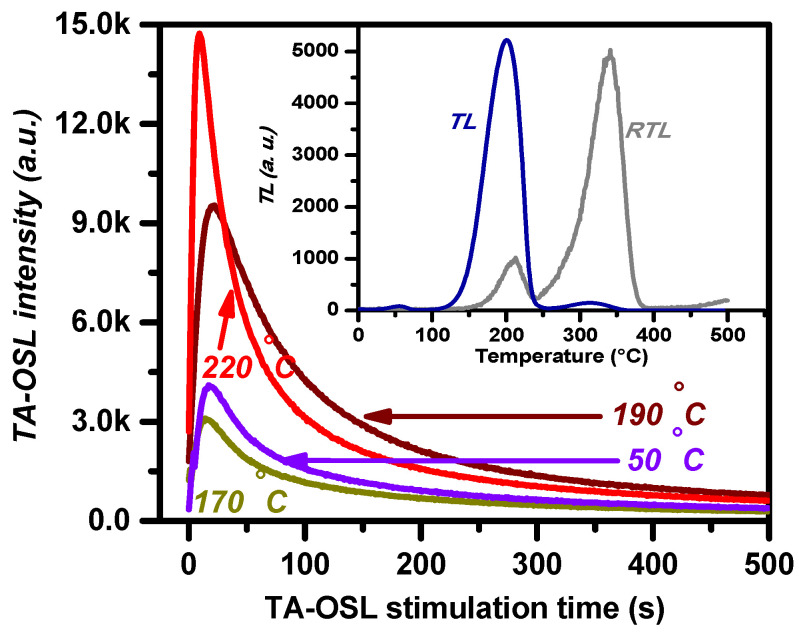
Examples of isothermal TA-OSL curves from BeO discs for four different measurement temperatures (protocol A); the inset presents a TL before and an RTL glow curve after TA-OSL at 190 °C. Intense TA-OSL signal is measured for all stimulation temperatures.

TL glow curves of BeO indicate the same features as those reported by Aşlar et al. [5]; these are ubiquitously present in all TL glow curves of both protocols (steps 3 and 8 in protocol A, steps 3, 5, and 11 in protocol B) in the present study, as the inset of Figure 1 reveals. Nevertheless, there is a shift of the *T_m_* towards higher values (203 and 329 °C for TL peaks 2 and 3, respectively). In the present study, the nomenclature will be the same as in the previous studies of our research group; the main dosimetric TL peak with delocalization temperature at 203 °C is termed TL peak 2 hereafter, while the TL peak with T_m_ = 329 °C is termed TL peak 3. Between these, TL peak 2 stands as the most intense, for a wide range of doses. For a detailed analysis on the kinetic parameters of the TL glow curves of this specific material, readers are referred to [5] and references therein.

**Figure 2:**
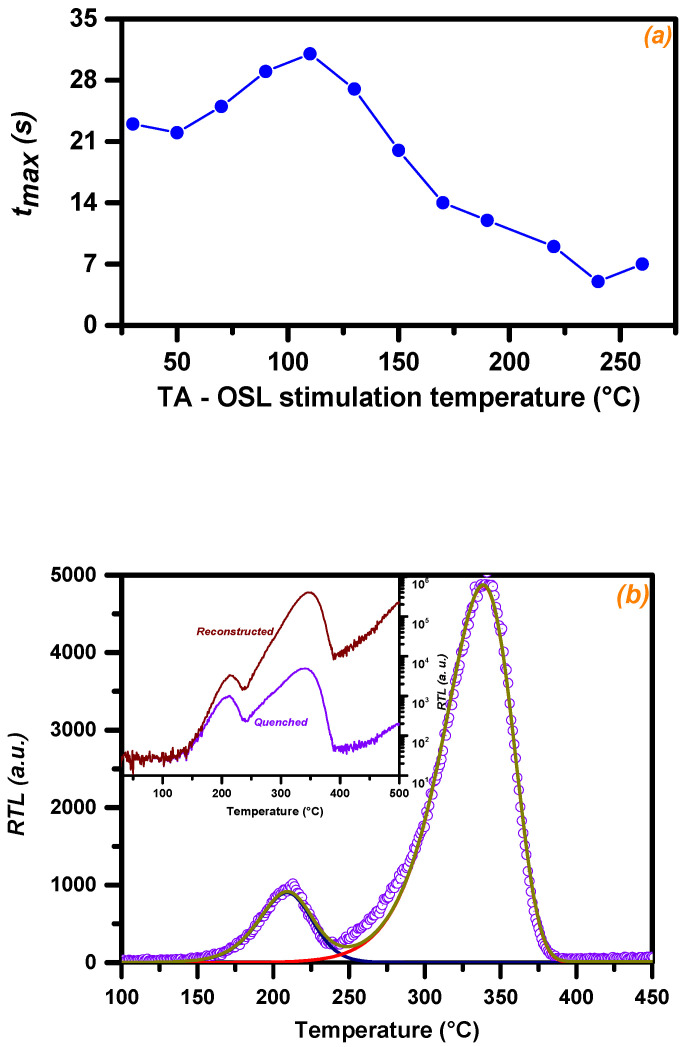
Plot (**a**) presents the time stimulation parameter *t_max_* corresponding to the maximum intensity of the isothermal TA-OSL curve from BeO discs versus different stimulation temperatures (protocol A). Plot (**b**) presents the RTL glow curve after TA-OSL at 190 °C (experimental points as open dots) deconvolved into its two individual TL peaks; solid lines correspond to each individual TL peak along with the fitted TL glow curve. The fitting parameters for each RTL peak stand in good agreement with the corresponding values of TL peaks 2 and 3. Inset of plot (**b**) presents the same RTL glow curve before and after reconstruction; TL signal for temperatures higher than 375 °C suggests the presence of at least one more TL peak with delocalization temperature beyond 500 °C.

Examples of TA-OSL curves from BeO for a selection of stimulation temperatures (Figure 1) include one at low temperature along with three at higher counterparts, namely *T_st_* = 50, 170, 190, and 220 °C. All curves correspond to TA-OSL measurements received promptly after the end of step 4 in protocol A. The signal in all cases emerges from VDTs, since each TA-OSL curve is monitored promptly after a TL measurement up to 500 °C. The shape of the TA-OSL does not resemble a classic background OSL signal. Instead, the intense TA-OSL signal is measured ubiquitously for all temperatures of stimulation according to step 5 of protocol A. All TA-OSL curves are bell-shaped, independent of the dose, similar to the TA-OSL curves reported for the case of aluminum oxide [20,21] and CaF_2_:N [41]. The maximum intensity of the TA-OSL is monitored at stimulation time *t_max_* (s); in general, this value is (a) lower than 50 s in all cases (thus, due to the 500 s of total stimulation, the peak-shaped TA-OSL signal yields a long tail at high stimulation times) and (b) shifted towards lower values as the stimulation temperature increases. Thus, as the *t_max_* is temperature-dependent, the bell-shaped TA-OSL curve changes with stimulation temperature. This feature is strongly revealed from Figure 2a; *t_max_* indicates an initial, wide peak with the maximum value attained at 110 °C, followed by an almost linear decrease for temperatures beyond. This behavior, being similar to the corresponding feature of the TA-OSL in aluminum oxide [20,21], provides an experimental argument for the presence of competition effects during TA-OSL recombination [42,43]. 

**Figure 3:**
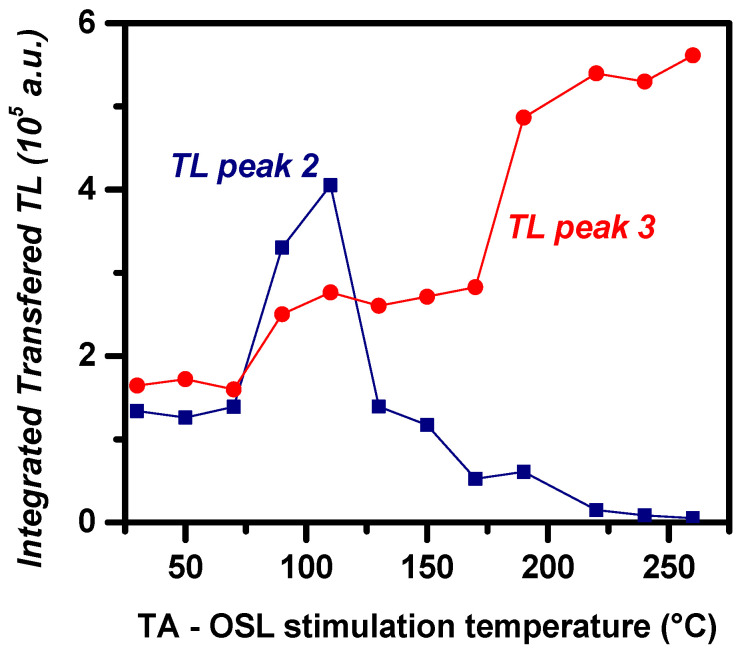
RTL signal (step 6 of protocol A) for both peaks 2 and 3 versus stimulation temperature of the TA-OSL signal (step 5), in terms of integrated TL signal following deconvolution analysis.

Residual TL (RTL) glow curves, namely TL signals promptly after the TA-OSL measurement, are measured during steps 6 and 9 in protocols A and B, respectively. RTL glow curves do not yield the unstable peak at ≈75 °C. Moreover, between TL peaks 2 and 3, RTL indicates the latter being more intense than the former for the majority of the stimulation temperatures. This feature is indicated by the inset of Figure 1, where both TL (from step 3) and RTL (from step 6) glow curves are presented for the case of the TA-OSL stimulation temperature of 190 °C. Figure 3 presents the intensities for both RTL peaks 2 and 3, plotted versus the TA-OSL measurement temperatures, in terms of integrated TL signal following deconvolution analysis. According to this figure, it becomes apparent that RTL peak 2 is more intense than RTL peak 3 only for a short range of stimulation temperatures (90 and 110 °C). Nevertheless, Figure 3 strongly supports the fact that intense transfer effects take place during the TA-OSL measurements. 

Within the temperature range 70–150 °C, intense transfer takes place to both RTL peaks; nevertheless, within this aforementioned temperature range, the transferred signal is much more intense for the case of RTL peak 2, as the stimulation temperature is lower than the delocalization temperature of this peak. For higher stimulation temperatures, which overlap with the temperature region of TL peak 2, intense transfer solely takes place to RTL peak 3. 

It is worth emphasizing the asymmetry of the shape of the latter RTL glow peak, implying the fact that it could be described by the first order of kinetics. The enhanced sensitivity of RTL peak 3 provides a unique opportunity to study the corresponding structure. All RTL glow curves from protocol A were deconvolved. The aim of this deconvolution is twofold, namely to (a) calculate the integrated TL signal for both peaks and use it to construct Figure 3, as well as (b) to calculate the kinetic parameters of both RTL peaks. As RTL peaks 2 and 3 are not overlapping, kinetic analysis for both was studied using both deconvolution as well as PSM. Figure 2b presents an example of a deconvolved RTL glow curve, while Table 3 presents an outline of the results of both the deconvolution as well as the PSM analysis concerning the activation energies and the order of kinetics for either RTL peak.

It is important to note that no correction for thermal quenching was applied before the RTL curves were analyzed using either the peak shape method or deconvolution analysis. The kinetic analysis on the RTL glow curves was performed to check whether the same trapping centers are responsible for the RTL and the ordinary TL signal. For such a comparison, reconstruction is not required. Moreover, Figure 2b presents an inset with an example of a reconstructed RTL glow curve. In both cases of reconstructed and quenched TL glow curves, the signal for temperatures beyond 375 °C justifies the presence of another TL glow peak with a delocalization temperature slightly larger than 500 °C. However, as this entire TL peak is not monitored, the deconvolution analysis of the entire TL glow curve will result in erroneous results due to resolution failure in the third peak. For a detailed analysis on the kinetic parameters of the reconstructed TL glow curves of this specific material, readers are referred to [5] and references therein.

### 3.2. TA-OSL versus Stimulation Temperature 

Measuring TA-OSL for various incrementally increasing stimulation temperatures has become a standard routine in isothermal TA-OSL studies of various materials, with the aim being threefold [6]: (a) to verify the thermally assisted nature of the signal, (b) to determine the activation energy of the thermal assistance of the procedure, and (c) to identify the optimum stimulation temperature *T***_st_** of isothermal TA-OSL.

**Figure 4:**
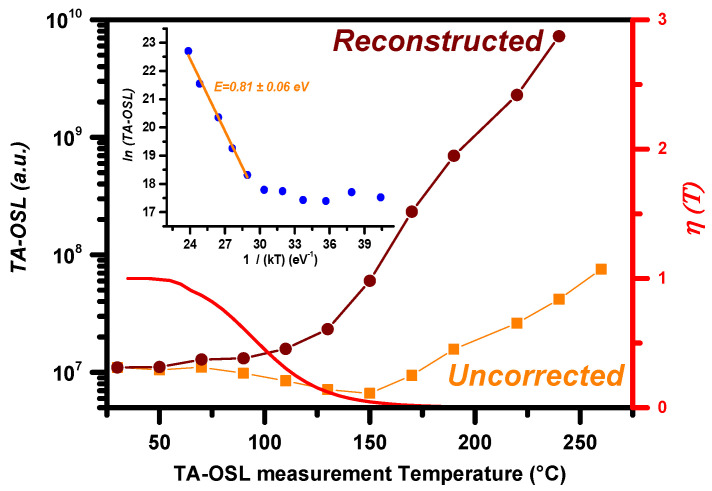
Integrated TA-OSL signals plotted versus the TA-OSL stimulation temperature (step 5 of protocol A). Both uncorrected data for thermal quenching as well as reconstructed data are presented. The same plot presents the thermal dependence of the thermal quenching efficiency *η*(*T*) (red line, right-hand axis). Inset presents the corresponding Arrhenius plot of the reconstructed data.

The integrated TA-OSL signal is plotted versus stimulation temperature in Figure 4. In all thermally assisted processes, unless thermal quenching is present, the signal intensity is expected to enhance with increasing stimulation temperature. Similar is the case for the thermally assisted OSL; the integrated OSL signal is expected to increase with increasing stimulation temperature, contrary to the signal of the conventional OSL, which is attributed to electrons from dosimetric traps. In the present case, the integrated TA-OSL signal (filled data points) indicates a smooth decrease for temperatures up to 170 °C, along with a mild increase for temperatures beyond. Nevertheless, these data were not corrected for the effect of thermal quenching. Figure 4 also presents the reconstructed data, namely following correction for compensation; these are presented as open data points. Such a correction was performed according to Equation (12). The behavior of the reconstructed data is similar to the behavior reported by Aşlar et al. [14] for the case of the component C_1_ of their ordinary OSL signal when measured at elevated temperatures (please refer to their Figure 12B).

For the calculation of the thermal assistance activation energy, the natural logarithm of the corrected (reconstructed) integrated TA-OSL signal is plotted versus the 1/kT (where T is the absolute stimulation temperature in units of K). The Arrhenius plot that is presented in the inset of Figure 4 is derived. A clear linear region is yielded in this latter Arrhenius plot. The slope of linear fit corresponds to the thermally assisted activation energy that was calculated as (0.81 ± 0.06) eV. This value stands among the highest activation energies calculated for the thermal assistance on the TA-OSL signal. Higher values were calculated and reported only for the cases of Al_2_O_3_:C (1.08 eV [20,21]) and quartz (0.95 eV [26]).

Finally, the main criterion for the selection of the optimum TA-OSL measurement temperature deals with an enhanced signal-to-noise ratio. Especially in the presence of thermal quenching, a TA-OSL signal with high intensity becomes crucial. According to Figure 4, TA-OSL measurement at temperatures higher than 190 °C is proposed; this is the temperature region at which the integrated TA-OSL signal increases monotonically with increasing temperature. For reasons that include technical limitation on the measurement instrumentation, the optimum temperature is 220 °C.

### 3.3. TL Sensitivity Changes following TA-OSL at Various Stimulation Temperatures 

**Figure 5:**
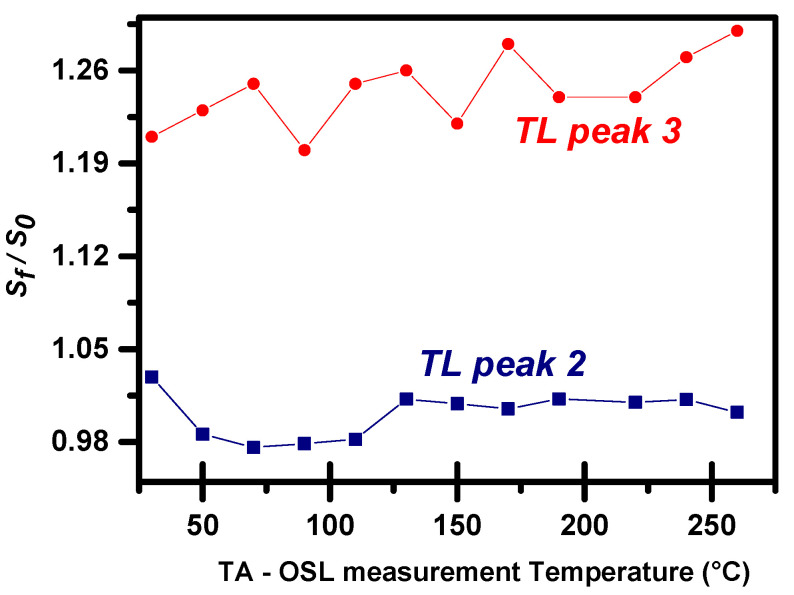
The ratio of the final (after measuring TA-OSL) over the initial (before measuring TA-OSL) TL sensitivity as a function of the stimulation temperature in the framework of protocol A.

Protocol A consists of eight steps for each stimulation temperature. Among these, four involve TL measurements. TL of step 1 is used just for zeroing purposes, while the variation of either the shape or the intensity of the RTL glow curve of step 6 has already been discussed in Section 3.1. The other two TL measurements are quite important, as the comparison between the *S_f_* and the *S*_0_ glow curves (before and after the TA-OSL measurement, respectively) provides strong indications regarding possible changes in either TL glow curve shape or sensitivity. Despite the fact that TA-OSL was reported to induce shape changes in many luminescence phosphors such as Al_2_O_3_:C [21] and halites [28], the shape of the TL glow curve of BeO does not change after TA-OSL measurement. Moreover, the corresponding ratio of $\frac{S_{f}}{S_{0}}$ provides for each TL peak a measure of sensitization; this plot is presented in Figure 5 for both TL peaks. It is important to note that this ratio takes a value around unity (within statistical errors) for TL peak 2. For TL peak 3, this latter ratio takes values varying between 1.2 and 1.3, implying mild sensitization by 20–30%. There is not any specific behavior of this ratio with the TA-OSL stimulation temperature. 

### 3.4. TL and TA-OSL Dose–Responses 

**Figure 6:**
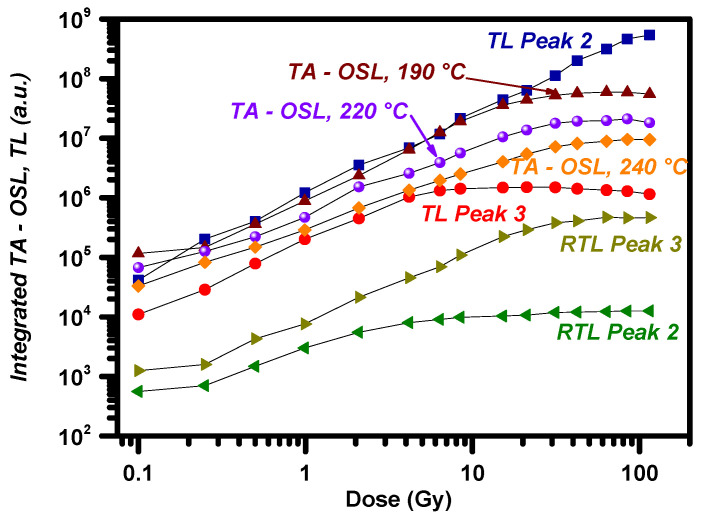
Dose–response curves in terms of integrated luminescence signals for TL and RTL glow peaks as well as TA-OSL signals presented in the same plot (protocol B).

While the application of protocol A is required from a methodological point of view, for the optimization of the TA-OSL measurement sequence, protocol B will provide the most important information from a dosimetric point of view. Figure 6 presents the dose–response curves for both TA-OSL and TL signals from BeO. This material indicates favorable TL dose–response features, as TL peak 2 yields supralinear dose response throughout three decades of doses, namely from 0.1 to 100 Gy. The dose response of TL peak 3 is also supralinear, indicating quick saturation around 10 Gy and beyond. These features stand in good agreement with the TL dose–response features that were presented by Polymeris et al. [15]; in this recent paper, the dose–response curves were fitted using the formula:

(13) $$TL,\;OSL=a\cdot D^{m}$$

with the linearity index *m* being 1.71 for the case of TL peak 2 and 1.45 for the case of TL peak 3. According to Section 3.2, the most appropriate temperature for the TA-OSL measurement is 220 °C. The corresponding TA-OSL dose–response curve is presented in Figure 6 in terms of integrated signal, indicating supralinearity up to almost 30 Gy and a saturation sublinearity for larger doses. Nevertheless, due to the relative low intensity of the integrated TA-OSL signal compared to the corresponding integrated signal of TL peak 2, the TA-OSL dose response was measured at two more temperatures, namely 190 and 240 °C; these temperatures were selected as they surround 220 °C. All dose–response curves are presented in Figure 6. By comparing these three TA-OSL doses–response curves, not only among them, but also to the corresponding counterparts of TL peaks 2 and 3, one can easily conclude that independent of the stimulation temperature, all integrated (over 500 s) TA-OSL signals are more intense than the integrated TL signal of peak 3. Moreover, the intensity of the TA-OSL at ***T_st_*** = 190 °C is almost of comparable magnitude to the integrated TL signal of peak 2. As the stimulation temperature increases, the integrated TA-OSL signal decreases, due to the effect of thermal quenching. Intense supralinearity features were yielded for all dose responses. Despite the fact that the dose responses are supralinear, no low-dose supralinearity is monitored for the TA-OSL at any stimulation temperature. Saturation sublinearity occurs for all three different stimulation temperatures. Moreover, as the stimulation temperature increases, so does the dose at which saturation takes place. Table 4 presents the saturation doses for each dose–response curve. 

Due to the large charge capacity of the VDTs, even after the end of the TA-OSL measurement of protocol B, a weak RTL signal is monitored. Two features are worth mentioning: (a) the shape of the RTL glow curve indicates similar features to the corresponding RTL of the previous protocol, namely the lack of the TL peak at 75 °C as well as the RTL peak 3 more intense than RTL peak 2, and (b) for both RTL peaks, the signal is almost stable within a wide dose region. Moreover, all RTL glow curves were subjected to both deconvolution and PSM analysis; the corresponding results are presented in Table 3. RTL integrated intensity versus initially attributed dose is also presented for both RTL peaks in Figure 6; it increases sublinearly with dose, while for larger doses it becomes stable and independent of dose. However, the integrated intensity is quite faint, almost three to four orders of magnitude lower than the TL and two to three less than the integrated TA-OSL signal. 

### 3.5. TL Sensitivity Changes following TA-OSL at Incremental Doses

**Figure 7:**
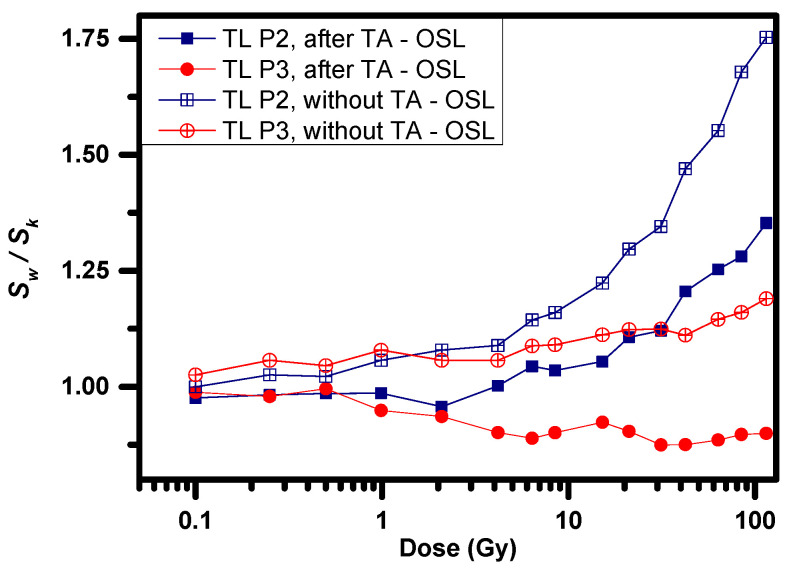
Sensitivity changes induced by the large dose accumulated (open data points) as well as by a combined action of large dose and a TA-OSL measurement (filled data points) for both TL peaks of BeO.

Protocol B was designed in such way to include one TL measurement following a fixed test dose of 0.1 Gy before (step 3, *S_k_*) and another one after the large dose and the TA-OSL (step 11, *S_w_*). By comparing these two aforementioned TL measurements, possible changes on either TL glow curve shape or sensitivity could be easily monitored. The first obvious result deals with the lack of any modification of TL glow curve shape over the entire dose region. On the contrary, mild sensitivity changes (or alternatively, sensitization) were registered (Figure 7, filled data points for both TL peaks 2 and 3). Nevertheless, this change of sensitivity could be attributed to the combined action of large dose accumulation and TA-OSL measurement. In order to check the impact of solely the large dose on these sensitivity changes, protocol B was applied once again without steps 7–9, namely without the application of TA-OSL measurement. 

These results are also presented in Figure 7 as open data points for both TL peaks. Insignificant sensitivity changes are monitored for doses as large as 1–5 Gy. For larger doses, mild sensitization is prominent for both TL peaks. This sensitization is dose-dependent, indicating an increasing trend with dose. This change in the TL sensitivity is larger in the absence of the TA-OSL measurement for both TL peaks. Moreover, between these two, sensitization is always larger for TL peak 2. 

## 4. Discussion

BeO, as a wide-band semiconductor, is another luminescent phosphor with very deep traps in its matrix. These traps could be effectively stimulated using TA-OSL, yielding an intense, bell-shaped signal. TA-OSL was used as an indirect verification for the presence of either very deep traps or transferred signal from these, solely by monitoring the increase in the signal versus increasing stimulation temperature. This feature is the main experimental evidence for a thermally assisted process and at the same time the main experimental difference between the TA-OSL and the conventional OSL signals. Of course, this increase was monitored even for the quenched signal, for stimulation temperatures above 170 °C. Nevertheless, following reconstruction, namely correction and compensation due to thermal quenching, this indication is prominent even from lower stimulation temperatures. According to [3,7,16,17,44], overlapping TL peaks were found to be located in the region 400–650 °C of the corresponding TL glow curve. For the case of the present study, the TA-OSL signal originates from TL traps with delocalization temperatures within the range from 500 to 650 °C. Moreover, both quenched but mostly the reconstructed RTL glow curves in the inset of Figure 2b provide strong experimental evidence supporting the existence of such traps. These delocalization temperatures are well below the corresponding temperatures for the VDTs of the Al_2_O_3_:C, with the latter being around 750–900 °C; thus, these traps could be easily stimulated via a direct TL measurement using the new luminescence instrumentation. Nevertheless, the interference from a high infrared background signal at such high temperatures, in conjunction with the thermal quenching effect, makes TL measurements up to temperatures as high as 600–650 °C quite problematic.

The presence of RTL signals in both TA-OSL protocols indicates that the main measurement in TA-OSL protocols is able to create PTTL by liberating electrons from VDTs. When PTTL is observed, then the TA-OSL possibly originates either directly from the VDTs or indirectly from the shallower trap, to which electrons are photo-transferred [6]. It is important to note that the RTL signal measured after the TA-OSL includes the afterglow emission due to radiative relaxation of unstable centers, in addition to the high-temperature blue stimulated OSL component. For the majority of the RTL glow curves, TL peak 3 not only exists, but also dominates in the RTL spectra after the step of TA-OSL. According to protocol A, the TA-OSL measurement temperature is always lower than the delocalization temperature of TL peak 3; nevertheless, it gets larger than the delocalization temperature of TL peak 2. Thus, during the TA-OSL measurement, the stimulation temperature is always lower than the T_m_ of TL peak 3. Therefore, the corresponding trap accumulates charge that recombines totally only during the following step of the RTL measurement.

The kinetic analysis that was performed in the RTL glow curves of both protocols provides solid proof regarding the dominance of the first order of kinetics in the mechanism of the transferred luminescence. This conclusion could imply a lack of re-trapping during the transfer effects. However, following a TA-OSL measurement for 500 s, the RTL signal registers the remaining electrons that were transferred to the main traps. These electrons are quite few while all VDTs are empty, inducing strong competition; the re-trapping probability is quite high and the number of re-trappings for every electron is large. According to Kitis and Pagonis [45], in such cases, the large number of re-trappings indicates TL glow curves that are also dominated by the first order of kinetics. The activation energies are slightly larger than 1 eV, in good agreement with the results of the activation energies reported by our group for both TL peaks 2 and 3 [4,5,14,15,40]. Moreover, following TA-OSL, there is no change in the TL glow curve shape of BeO; such a change was quite obvious in both cases of Al_2_O_3_:C and NaCl. All aforementioned features stand as arguments for the fact that TL, PTTL, and TA-OSL use the same traps, being more than one, in addition to the main dosimetric trap that is responsible for TL peak 2. 

The bell peak shape of the TA-OSL signal is quite important, as it can provide strong hints for the identification of the recombination pathways of TA-OSL. The presence of the peak-shaped isothermal TA-OSL curve, in conjunction with a strong PTTL signal, could be considered as being typical of transfer effects taking place via the conduction band. This conclusion is further supported by the model adopted for the fitting of these curves in the case of CaF_2_:N [41]. According to a recent paper by Polymeris et al. [46], the bell shape is typical for the cases where recombination takes place via the conduction band. This was verified using Time-Resolved TA-OSL measurements for the case of Al_2_O_3_:C [47]; TR–TA-OSL measurements will provide stronger arguments for the case of BeO as well. The shape of the TA-OSL curves could be used as a probe in order to distinguish the recombination pathways of these signals. 

As far as the dosimetric properties of BeO’s TA-OSL are concerned, the present study revealed that the TA-OSL signal could be effectively used for dosimetry purposes. In fact, independent of the stimulation temperature, the dose response of the TA-OSL signal indicates supralinearity features, similar to the counterparts of either TL or the component C_2_ of the ordinary OSL signal [15]. Unfortunately, the capacity of these traps in BeO is not high enough to enable extension of the detectable dose limit of the material beyond 35–67 Gy, being the current saturation doses of the TA-OSL, depending on the stimulation temperature. Furthermore, the dose response of the (main dosimetric) TL peak 2, despite being supralinear, extends the detectable dose up to almost 100 Gy. Thus, the dosimetric properties of the TA-OSL signal are not as favorable for being exploited for high-dose-level dosimetry purposes as was suggested for Al_2_O_3_:C [20,21]. 

Nevertheless, the results have obvious implications for practical dosimetry using BeO. As was also the case for TT-OSL [3,16], TA-OSL also enables re-estimation of the doses in order to perform a cross-check in cases where the detectors are accidentally exposed to light. Moreover, the standard procedure for measuring doses using BeO includes a short optical stimulation that is followed by an intense optical zeroing of the optically sensitive traps. As the photo-ionization cross-section of the VDTs is quite small and these traps are slightly affected by light at ambient temperatures, in a series of consecutive and repetitive dose estimations using the same BeO disk, TA-OSL could be effectively used for the calculation of the cumulative dose over the entire number of consecutive uses. Experimental studies on BeO crystals have revealed several features which do not follow from the known models describing the luminescence kinetics in solids. Such features include supralinear dose responses along with sensitivity changes of both TL dosimetric peaks; these features are enhanced following long-term use of the same BeO disk due to the large accumulated dose to the VDTs. Even though these features are still not thoroughly understood, our experience gained from similar studies on Al_2_O_3_:C indicated that a considerable advancement in the understanding of the mechanism of these observed phenomena was the establishment of the participation of very deep traps in these processes [48,49,50]. The occupancy of the VDTs, a measure of which could be provided by the cumulative dose accumulated in them, could be the most important parameter affecting these features. The occupancy of VDTs is controlled by selectively emptying them using TA-OSL measurements. The less occupied these traps are, the more intense competition effects take place. The present study has revealed that TA-OSL is able to limit the sensitivity changes that might be induced due to long-term uses and large accumulated doses to the very deep traps. 

## 5. Conclusions

TA-OSL is an alternative experimental method to not only measure the signal from very deep traps in BeO without heating the sample to temperatures greater than 500 °C, but also exploit the properties of this signal for dosimetric purposes as well. The intense, peak-shaped TA-OSL signal is measured throughout the entire temperature range. This signal is attributed to intense transfer effects from VDTs to both TL peaks 2 (main dosimetric) and TL peak 3. The increase in the integrated signal for increasing stimulating temperatures indicates the thermally assisted nature of the signal. A temperature of 220 °C was selected as the optimum stimulation temperature, mostly for signal-to-noise ratio reasons. The dose response of the TA-OSL integrated signal is sublinear, indicating practically saturation levels at relatively low doses. Isothermal TA-OSL is not effective for extending the maximum detection dose thresholds of BeO. Nevertheless, TA-OSL could be effectively used in order to either (a) control the occupancy of VDTs and thus get rid of intense sensitivity changes induced by long-term uses and high accumulated dose to the VDTs or (b) measure the dose accumulated over a series of repetitive dose calculations. 

## Figures and Tables

**Table 1:** Protocol A: TA-OSL for various stimulation temperatures for identifying the optimum stimulation temperature. The sample was cooled down to room temperature at the end of step 5 of each measurement cycle.

Step No.	Action	Comments and Technical Specifications
Step 1:	TL measurement	*Remove any prior existing signal*
Step 2:	Irradiation using 5 Gy dose	*Populate all traps, including the VDTs*
Step 3:	TL measurement	*Empty shallow, dosimetric, and deep traps and monitor initial sensitivity S_0_*
Step 4:	Isothermal TL (ITL) (at room temperature for 60 s)	*Check for possible overflow of the PMT*
Step 5:	OSL measurement at temperatures ***T_st_*** ranging from ***30 *°*C*** up to ***270 *°*C*** (in steps of 20 °C) over a period of 500 s	*Measure the TA-OSL curves for various stimulation temperatures*
Step 6:	TL measurement	*Obtain the Residual TL* (*RTL*) *curves*
Step 7:	Irradiation using 5 Gy dose	*Populate all traps*
Step 8:	TL measurement	*Monitor final sensitivity S_f_*

**Table 2:** Protocol B: TA-OSL for various doses to check the dose–response behavior of the signal. The sample was cooled down to room temperature at the end of step 7 of each measurement cycle.

Step No.	Action	Comments and Technical Specifications
Step 1:	TL measurement	*Remove any prior existing signal*
Step 2:	Irradiation using 0.1 Gy dose	*Populate shallow, dosimetric, and deep traps using the minimum dose*
Step 3:	TL measurement	*Empty shallow, dosimetric, and deep traps and monitor initial sensitivity S_k_*
Step 4:	Irradiation with a dose *D_i_* *	*Populate all traps, including the VDTs*
Step 5:	TL measurement	*Empty shallow, dosimetric, and deep traps and monitor high-dose sensitivity S_d_*
Step 6:	Isothermal TL (ITL) (at room temperature for 60 s)	*Check for possible overflow of the PMT following the TL measurement*
Step 7:	OSL measurement at the optimum stimulation temperature ***T_st_*** over a period of 500 s	*Measure the TA-OSL curves for various doses*
Step 8:	Isothermal TL (ITL) (at room temperature for 60 s)	*Check for possible overflow of the PMT following the TA-OSL measurement*
Step 9:	TL measurement	*Obtain the Residual TL* (*RTL*) *curves*
Step 10:	Irradiation using 0.1 Gy dose	*Populate shallow, dosimetric, and deep traps using the minimum dose*
Step 11:	TL measurement	*Monitor final sensitivity S_W_*

* The doses applied were 0.1, 0.25, 0.5, 1, 2, 4, 6.5, 8.5, 15, 21, 32, 43, 65, 85, and 110 Gy; each cycle of steps 1–11 was applied to different BeO disk.

**Table 3:** Kinetic parameters of the RTL glow peaks from both protocols according to the peak shape method (PSM) and deconvolution analysis. All symbols were explained inside the text and correspond to averages over the number of existing RTL glow curves for each protocol; ***E****_Dec_* signifies the activation energy that was calculated according to the deconvolution analysis, while ***E****_PSM_* is the respective value according to peak shape method analysis.

Protocol	RTL Peak *T_m_* (°C)	*E_Dec_* (eV)	*R*	*ω* (°C)	*E_PSM_* (eV)	*b*
A	203.0 ± 1.0	1.21 ± 0.14	0.11 ± 0.03	43.0 ± 1.0	1.09 ± 0.09	1.15 ± 0.06
A	329.5 ± 0.5	1.32 ± 0.12	0.09 ± 0.02	54.0 ± 0.5	1.16 ± 0.11	1.09 ± 0.04
B	202.0 ± 1.5	1.18 ± 0.15	0.15 ± 0.02	41.5 ± 1.5	1.14 ± 0.12	1.14 ± 0.08
B	328.5 ± 1.5	1.27 ± 0.15	0.11 ± 0.01	54.5 ± 0.5	1.20 ± 0.12	1.07 ± 0.05

**Table 4:** Saturation doses according to the corresponding dose–response curves.

Lum. Signal	TL Peak 2	TL Peak 3	TA-OSL, 190 °C	TA-OSL, 220 °C	TA-OSL, 240 °C	RTL Peak 2	RTL Peak 3
Saturation Dose (Gy)	-	13 ± 1	35 ± 3	52 ± 3	67 ± 4	7 ± 1	42 ± 4

## Data Availability

Data are available upon request.
